# Three-D Wide Faces (3DWF): Facial Landmark Detection and 3D Reconstruction over a New RGB–D Multi-Camera Dataset

**DOI:** 10.3390/s19051103

**Published:** 2019-03-04

**Authors:** Marcos Quintana, Sezer Karaoglu, Federico Alvarez, Jose Manuel Menendez, Theo Gevers

**Affiliations:** 1 Grupo de Aplicación de Telecomunicaciones Visuales, Universidad Politecnica de Madrid, 28040 Madrid, Spain; fag@gatv.ssr.upm.es (F.A.); jmm@gatv.ssr.upm.es (J.M.M.); 2 Computer Vision Lab, University of Amsterdam, 1098 XH Amsterdam, The Netherlands; s.karaoglu@uva.nl (S.K.); th.gevers@uva.nl (T.G.); 3 3DUniversum, 1098 XH Amsterdam, The Netherlands

**Keywords:** face landmark detection, 3D face modelling, head pose classification, 3D data collection, deep learning

## Abstract

Latest advances of deep learning paradigm and 3D imaging systems have raised the necessity for more complete datasets that allow exploitation of facial features such as pose, gender or age. In our work, we propose a new facial dataset collected with an innovative RGB–D multi-camera setup whose optimization is presented and validated. 3DWF includes 3D raw and registered data collection for 92 persons from low-cost RGB–D sensing devices to commercial scanners with great accuracy. 3DWF provides a complete dataset with relevant and accurate visual information for different tasks related to facial properties such as face tracking or 3D face reconstruction by means of annotated density normalized 2K clouds and RGB–D streams. In addition, we validate the reliability of our proposal by an original data augmentation method from a massive set of face meshes for facial landmark detection in 2D domain, and by head pose classification through common Machine Learning techniques directed towards proving alignment of collected data.

## 1. Introduction

Recent advances in computer vision and machine learning have directed the research community towards building large collections of annotated data in order to increase the performance of different application domains. In an analogous manner, 3D imaging has arrived by the introduction of low-cost devices and the development of efficient 3D reconstruction algorithms. In this paper, we focus on 3D facial imaging. Facial attributes range from simple demographic information such as gender, age, or ethnicity, to the physical characteristics of a face such as nose size, mouth shape, or eyebrow thickness, and even to environmental aspects such as lighting conditions, facial expression, and image quality [1].

The first attempt to collect facial data to study faces is through the introduction of Multi-PIE database [2], where the facial appearances vary significantly by a number of factors such as identity, illumination, pose, and expression. They built a setup with 13 cameras located at head height and spaced over 15 intervals. Multi-PIE database contains 337 subjects, imaged under 15 view points and 19 illumination conditions. This work is the starting point of the benchmark 300-W [3] that establishes the main metrics and standards to normalize the evaluation of the different methods for facial landmark analysis. One of the standards established by 300-W is the annotation of facial landmarks.

More recently, 2D traditional approaches for facial analysis have been using low-cost depth sensing devices by providing 3D modelling data sources. An important topic today is 3D face modeling employing RGB–D sensing devices. Traditional works mostly focus on providing accurate 3D face models. Recently, it was demonstrated that laser scan quality face models can be reconstructed from RGB–D sequences [4,5].

Head pose estimation is another important but challenging task for face analysis. Head pose provides important meta-information about communicative gestures, salient regions in a scene based on focus of attention, and can be used in surveillance environments to perform behaviour analysis [6,7].

It is known that deep learning methods are data hungry. Hence, there exists a data-demanding problem of deep networks, and tedious data annotation procedures related. This data problem can be solved by generating synthetic data [8,9]. In this paper, we have employed 3D meshes of 300 faces of subjects from 3DU dataset that allows for a proper augmentation method whose parameters are Euler angles [10] (pitch, yaw and roll). With this method, we only need to annotate every model once, but then we can generate thousands of augmented images for the different head poses.

The contributions of this work are:

1. **3DWF** We propose a multi-camera dataset containing visual facial features. We include streams from 600 to 1200 frames of RGB–D data from three cameras for 92 subjects and clouds normalized to 2 K points for ten poses proposed and their corresponding 3D landmarks projected. Demographic data such as age or gender is provided for every subject as well. Such a complete dataset in terms of the number of subjects and different imaging conditions is unique and the first of its kind.
2. An innovative **data augmentation method for facial landmark detection**. This method is based on a 3D(mesh)-2D projection implemented by raycasting [11].
3. **3D reconstruction workflow** adapted to facial properties and their normalization to provide meaningful features for cloud formats.

The paper is organized as follows:

1. **Section 2** outlines the techniques related to the work presented here.
2. **Section 3** presents the set-up for the acquisition of the proposed dataset.
3. **Section 4** introduces an innovative data augmentation for facial landmark detection.
4. **Section 5** describes a complete pipeline for 3D face modeling with a multi-camera RGB–D setup.
5. **Section 6** presents a validation method for the classification of the markers.
6. **Section 7** validates the captured data and evaluates the proposed methods for facial landmark detection.
7. The main contributions are discussed in **Section 8**.

## 2. Related Work

This section describes the current algorithms related to facial analysis that make use of computer vision techniques. We focus on the following facial analysis applications: 3D facial acquisition, facial landmarks detection, and head pose estimation.

### 2.1. 3D Facial Acquisition

Recently, different approaches have been published to optimize acquisition systems to accurately represent 3D facial attributes. Ref. [12] collected 2000 2D facial images of 135 subjects as well as their 3D ground truth face scans. The authors proposed a dense face reconstruction method based on dense correspondence from every 2D image gathered to a collected neutral high resolution scan. Another hybrid solution (Florence Dataset) for reconstruction is presented in [13], where a complex capturing system (3dMD [14]) is used. The number of subjects (53) is also smaller than the one proposed here. UHDB31 [15] presented a follow-up of this work by increasing the number of poses and the number of subjects resulting in a more complete dataset, but still costs of the set up are quite high and the number of subjects are still under the ones captured in this work.

In our case, we provide neutral high resolution scans as well, but we believe that the impressive results of recent works published, implementing deep learning for classification and segmentation with normalized input clouds [16,17] motivate a new research line. Therefore, we postulate a new challenge, and propose an initial reconstructed and normalized set to adopt this line for facial analysis.

The Pandora dataset, focusing on shoulder and head pose estimation, is introduced in [18] for driving environments. However, the dataset only contains images from 20 subjects and they only have one camera, and therefore it does not allow proper 3D reconstruction for extreme poses. Ref. [19] proposes a 3D reference-free face modeling tested on a set of predefined poses. The authors perform an initial data filtering process, and employ the face-pose to adapt the reconstruction. In our case, we use 2D face detection projection, and afterward implement the proper 3D filtering techniques, exploiting information from 2D facial landmark detection in order to perform a more reliable 3D face reconstruction. Other techniques are proposed by simply using a single RGB sensor, but in this case they require either a 3D Morphable Model (3DMM) initially proposed by Blanz and Vetter [20], this kind of method can be trapped in a local minimum and can not generate very accurate 3D geometry, or a 2D reference frame and displacement measurement, as in [21].

### 2.2. Facial Landmark Detection

Research in this field is very proficient. Therefore, we concentrate on the three groups most relevant to our work:

1. **Regression-Based Methods**. These methods directly learn a regression function from image appearance (feature) to the target output (shape):
  (1) $$M:F\left(I\right)\to x\in R^{2N}$$
  where *M* denotes the mapping from an image appearance feature (F(I)) to the shape *x*, and *F* is the feature extractor.
  Ref. [22] proposed a two-level cascaded learning framework based on boosted regression. This method directly learns a vectorial output for all landmarks. Shape-indexed features are extracted from the whole image and fed into the regressor.
2. **Graphical Model-based Methods**. Graphical model-based methods mainly refer to tree-structure-based methods and Markov Random Field (MRF) based methods. Tree-structure-based methods take each facial feature point as a node and all points as a tree. The locations of facial feature points can be optimally solved by dynamic programming. Unlike the tree-structure that has no loops, MRF-based methods model the location of all points by loops.
  Zhu and Ramanan [23] proposed a unified model for face detection, head pose estimation and landmark estimation. Their method is based on a mixture of trees, each of them corresponds to one head pose view. These different trees share a pool of parts. Since tree-structure-based methods only consider the local neighboring relation and neglect the global shape configuration, they may easily lead to unreasonable facial shapes.
3. **Deep Learning-Based Methods**. Luo et al. [24] proposed a hierarchical face parsing method based on deep learning. They recast the facial feature point localization problem as the process of finding label maps. The proposed hierarchical framework consists of four layers performing respectively the following tasks: face detector, facial parts detectors, facial component detectors and facial component segmentation.
  Sun et al. [25] proposed a three-level cascaded deep convolutional network framework for point detection in a coarse-to-fine manner. It can achieve great accuracy, but this method needs to model each point by a convolutional network that increases the complexity of the whole model. Ref. [26] enhanced the detection by following a coarse-to-fine manner where coarse features inform finer features early in their formation, in such a way that finer features can make use of several layers of computation in deciding how to use coarse features. We selected this method to test the data augmentation method presented in Section 4.1 because of the novelty and efficiency of a deep net that combines convolution and max-pool layers to train faster than the summation baseline and yields more precise localization predictions.
  Finally, the other selected solution to test the performance of the proposed augmentation method (Section 4.1) is [27] as they imply an evolution from previous models. As the proposed Tweaked Neural Network does not involve multiple part models, it is naturally hierarchical and requires no auxiliary labels beyond landmarks. They provide an analysis of representations produced at intermediate layers of a deep CNN trained for landmark detection, yielding good results at representing different head poses and (some) facial attributes. They inferred from previous analysis that the first fully connected layer already estimates rough head pose. With this information they can train pose specific landmark regressors.

### 2.3. Head Pose Estimation

Head pose estimation is a topic widely explored with applications such as autonomous driving, focus of attention modeling or emotion analysis. Fanelli et al. [28] introduced the first relevant method to solve this problem relying on depth sensing devices. Their proposal is based on random regression forests by formulating pose estimation as a regression problem. They synthesize a great amount of annotated training data using a statistical model of the human face. In an analogous way, Liu et al. [8] also propose a training method based on synthetic generated data. They use a Convolution Neural Network (CNN) to learn the most relevant features. To provide annotated head poses in the training process, they generate a realistic head pose dataset through rendering techniques. They fuse data from 37 subjects with differences in gender, age, race and expression. Some other lines of research, such as the one followed by [29], pose the problem as the classification of human gazing direction. We follow this approach in our work. It proposes as well deep learning techniques to fuse different low resolution sources of visual information that can be obtained from RGB–D devices. The authors encode depth information by adding two extra channels: surface normal azimuthal and surface normal elevation angle. Their learning stage is divided into two CNNs (RGB and depth inputs). The information learned by deep learning is employed to further fine-tune a regressor.

Analyzing all literature related to our work, we can conclude that our multi-camera RGB–D setup provides an affordable capturing system, able to perform 3D face reconstruction at extreme poses with a reasonable cost and deployment. In addition, facial landmark detection is already been explored extensively. Therefore, it is more suitable to provide a refinement of the 3D techniques being presented here. Head pose estimation is highly correlated with facial landmark detection (especially in 3D domain) and we believe with a good performance in facial landmarks head pose could easily be approached.

## 3. 3DWF Dataset

This dataset is captured by a system composed of 3 Asus Xtion depth cameras [30] in order to acquire multi-camera RGB–D information from 92 subjects by modifying their head poses steered by a sequence of markers. The subjects were asked to move their head continuously in a natural manner. To achieve a synchronous acquisition with three simultaneous devices, three independent USB buses are required, and synchronization among them has been implemented to provide a uniform acquisition. Synchronization among the devices is very critical since subjects are moving their head, and reducing delay between the cameras allows registration of the point clouds acquired. For that aim OpenNI 2 library [31] has been adopted by following the following procedure:

1. List of connected devices is gathered.
2. Every sensor is activated.
3. Data structures required to perform data flow are created.

**Figure 1:**
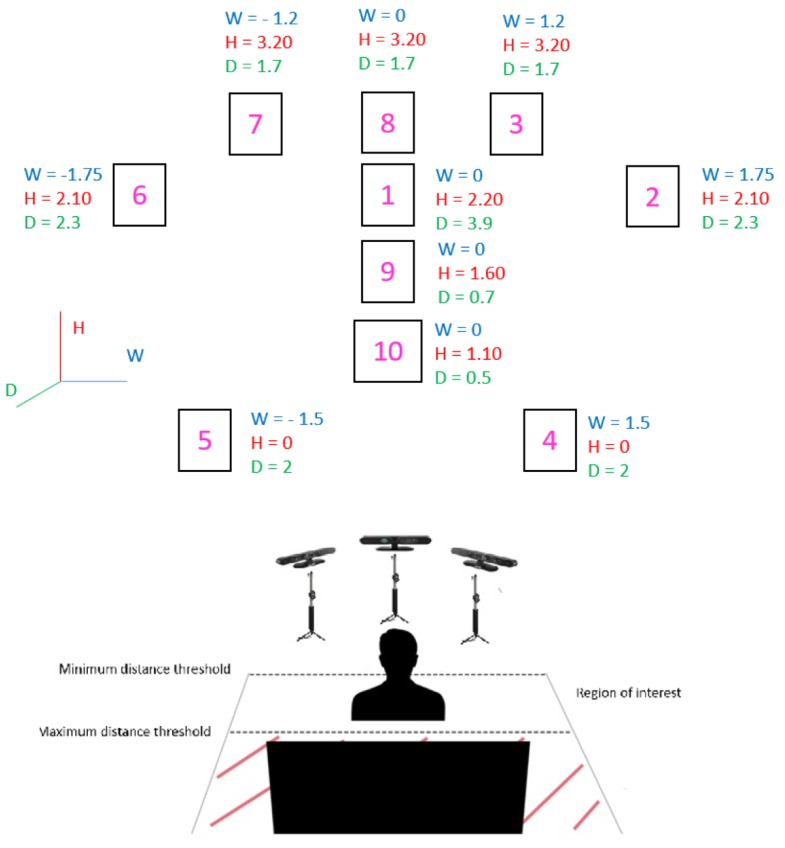
Graphical description of the proposed scenario to capture the 3DWF dataset. Coordinates of the markers are expressed in meters.

First generation of RGB–D sensors are deployed due to their higher accuracy to perform 3D reconstruction of one category of objects proved in [32], and their feasibility to connect more than one device to the same computer. Between 600–1200 frames are recorded by each device for every subject. The proposed setup is displayed in Figure 1. Three RGB–D cameras can be observed in the Figure 1, the one in the middle will be named as frontal camera, and the other ones as side cameras. The number in the box represents the sequence of markers that the subjects were asked to follow (starting in box 1 and ending in box 10). Where *W* stands for width, *H* for height and *D* for depth, and origin is located at the frontal camera for *W* and *D*, and the floor for *H*. The proposed dataset contains the following sources:

1. Visual data
  (a) RGB and Depth data. This data has been continuously captured and is relevant for topics such as facial tracking or 3D face reconstruction.
  (b) RGB point clouds for ten markers. This data has been statically reconstructed with a target resolution of 2K, and it is very suitable for machine learning methods related to the tasks such as head pose or gaze estimation.
  (c) HD initial cloud. This data can be useful as reference cloud and has been captured with Faro Freestyle 3D Laser Scanner [33] whose 3D point accuracy is 0.5 mm and reconstructed by FARO Scene [34].
2. Subject data
  (a) Age
  (b) Gender

### 3.1. Set-Up Optimization

Optimization tests are mainly based on three parameters:

1. **Distance from the model to the frontal camera.** The manufacturer of the device recommends a distance in the range of 80–150 cm. Therefore, the tests are performed in this range. From Table A2, it can be derived that the highest number of points in the point cloud are obtained with distances of 80 cm. Analogously, the best visual appearance is gathered with that value.
2. **Light source.** Once the optimum distance from the model to the camera is calculated, the next step is to determine the parameters related to the LED light source employed whose main features are expressed in Table A1. Different tests are performed based on three parameters:
  (a) Distance.The tests performed for the distance were mainly based on visual appearance in the cloud obtained. For distances smaller than 200 cm appearance was too bright. We found that optimum distance should be set to 250 cm.
  (b) Luminous flux. We base our evaluation on the resolution of the pointcloud obtained for each camera. Results obtained can be noted in Table A3. It can be derived that as long as the luminous flux increases, the resolution of the point cloud decreases. Therefore, 250 Lumens (lm) (minimum provided by the manufacturer) is chosen.
  (c) Orientation. To optimize orientation of the light source at the proposed scenario, grayscale mean ($\hat{I}$) and Standard deviation ($\sigma_{I}$) values are evaluated. To this end, different angles between the light sources are explored with a luminous flux of 250 lm, but in this case, we also should consider the visual appearance. For that purpose, we have tested light sources pointing to three targets:
    - Models
    - Frontal cameras
    - Side cameras
    Tests performed pointing to the models presented the worst visual results, even though different diffusion filters have been tested on the light source. Other values are shown in Table A4. The best visual results were obtained when light sources were pointing to the opposite side cameras. In this case, the mean and STD are replaced by the median ($\tilde{I}$) and Median Absolute Deviation (MAD), since the median and the mean own a notable difference. Also angles of the cameras are included in the table since they have a large influence on the results. Further, this parameter will be analyzed (separately) below.
3. **Camera orientation.** To test the best orientation of the cameras, different angles are used. All optimum parameters exposed previously are deployed in the scenario to test this parameter. The RGB–D devices chosen capture the scene affected by all parameters previously exposed, and therefore we believe it might be last parameter to be tested, and most critical since it determines the field of view. First tests are carried to determine the region of interest to be covered for the subjects involved in the experiment. The minimum angle required to completely cover the face of the subjects is $30^{\circ }$ and as long as the angles among the cameras are increased, surface covered increases as well. Finally, grayscale values obtained by the RGB sensor are evaluated to capture a similar range of color intensities for the faces. Results are shown in Table A5. It can be derived that as long as the angle among the cameras is increased, the difference between mean and the STD is also increased. Therefore, we can conclude that the optimum angle between the cameras is $30^{\circ }$.

### 3.2. Subjects Description

**Figure 2:**
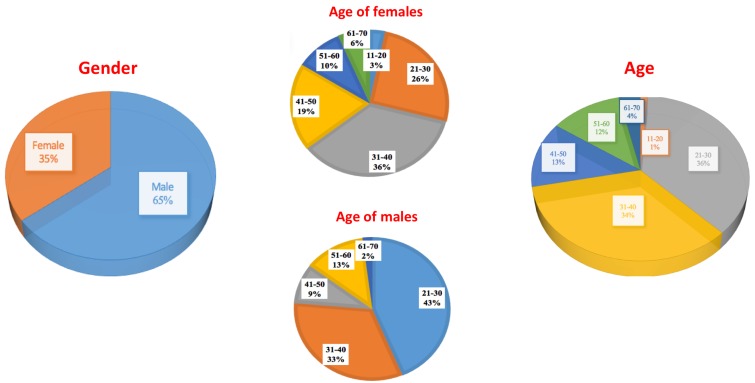
Graphics showing the most important features of the subjects included in 3DWF dataset: age and gender.

For this dataset, age and gender are also registered for all the subjects. Statistics of those features are shown in Figure 2.

We can observe that most of the population is located between 20 and 40 years old due to the fact that the dataset has been recorded in a university, but the dataset covers a wide range of ages. It is also noticeable that gender is a little unbalanced, however, looking to the specific graphs of age ranges for every gender it can be observed that age of females is more balanced than the age of males.

## 4. Facial Landmark Detection

This section proposes a new data augmentation method from 3D meshes to 2D images and analyzes its influence on two state of the art deep learning facial landmark detection methods.

### 4.1. Data Augmentation

**Figure 3:**
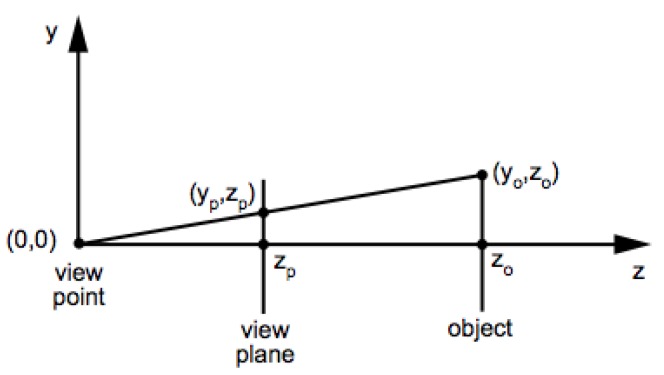
Raycasting geometry model with a plane and a pinhole. Extracted from [11] and reproduced with permission from Prof. House.

With the data augmentation method proposed for face landmark detection we wanted to prove a possible application for the dataset proposed (3DWF). The dataset provided by 3DUniversum is captured by a rotating structured sensor device in order to reconstruct 3D models of 300 subjects. Rotation is performed by an analogous device to the one presented in [35], however, in this case projector is not required, and structured light sensor is combined with common RGB sensor to perform the capture. This dataset was gathered by performing a massive data collection which allowed to collect a larger number of subjects, in spite of collecting less facial attributes such as pose, gender of age from every one of them, 3D reconstruction of gathered data is outside of the scope of this work. In our case, we are directed towards a deep learning method to extract valuable features from meshes already processed. To this end, we used raycasting [11] to perform the projection of the 3D mesh to a 2D image. By implementing this technique, we moved the viewing plane in front of the pinhole to remove the inversion. A graphical explanation is shown in Figure 3. If an object point is at distance $z_{0}$ from the viewpoint, and has *y* coordinate $y_{0}$, then its projection $y_{p}$ onto the viewplane is determined by the ratios of sides of similar triangles: $\left(0,0\right),\left(0,z_{p}\right),\left(y_{p},z_{p}\right)$, and $\left(0,0\right),\left(0,z_{0}\right),\left(y_{0},z_{0}\right)$. So we have:

(2) $$\frac{y_{p}}{z_{p}}=\frac{y_{0}}{z_{0}}$$

The values of the viewpoint are based on the following parameters and values:

1. **Pitch.**$\left[-30^{\circ },30^{\circ }\right]$ Interval: $5^{\circ }$
2. **Yaw.**$\left[-30^{\circ },30^{\circ }\right]$ Interval: $5^{\circ }$
3. **Roll.**$\left[-30^{\circ },30^{\circ }\right]$ Interval: $5^{\circ }$
4. **Distance.**[110 cm,160 cm] Interval: 10 cm

Subjects are split in three datasets following the deep learning paradigm.

1. Training set: 245 subjects (81.67%).
2. Validation set: 40 subjects (13.34%).
3. Testing set: 15 subjects (5%).

### 4.2. Deep Learning Architectures

#### 4.2.1. VanillaCNN

The solution shown in [27] is selected as one suitable architecture to increase the performance of facial landmark detection for Annotated Facial Landmarks in the Wild Dataset (AFLW [36]) based on data augmentation method previously discussed. The architecture of this network includes mid-network features and implies a hierarchical learning. The main peculiarity of this network is the tweaking model oriented to two main processes:

1. It performs a specific clustering in the intermediate layers by a representation that discriminates between differently aligned faces. With that information, it trains pose specific landmark regressors.
2. The remaining weights from the first dense layer output are fine-tuned by selecting only the group of images classified in the same cluster with the features from the intermediate layers.

An absolute hyperbolic tangent is used as an activation function and Adam is used for training optimization [37]. L2 normalized by the inter-ocular distance is implemented as the network loss:

(3) $$\varphi \left(P_{i},{\hat{P}}_{i}\right)=\frac{∥P_{i}-{\hat{P}}_{i}{∥}_{2}^{2}}{∥{\hat{p}}_{i,1}-{\hat{p}}_{i,2}{∥}_{2}^{2}}$$

where $P_{i}$ is the 2x*k* vector of predicted coordinates for a training image, ${\hat{P}}_{i}$ their ground truth locations, and ${\hat{p}}_{i,1}$, ${\hat{p}}_{i,2}$ is the reference eye position.

#### 4.2.2. Recombinator Networks (RCN)

Ref. [26] performs learning through using landmark independent feature maps. In this case, instead of performing specific learning, a more purely statistical approach is performed. The output of each branch is upsampled, then concatenated with the next level branch with one degree of finer resolution. Therefore, the main novelty is that branches pass more information to each other during training letting the network learn how to combine them non-linearly to maximize the log likelihood of the landmarks. It is only at the end of the Rth branch that feature maps are converted into a per-landmarks scoring representation by implementing a softmax.

All convolutional layers are followed by ReLU non-linearity except for the one right before the softmax. This architecture is trained globally using gradient backpropagation with an additional regularization term for the weights calculated through the next equation:

(4) $$\begin{array}{c} L\left(W\right)=\frac{1}{N}\sum_{n=1}^{N}\sum_{k=1}^{K}-logP\left(Y_{k}=y_{k}^{\left(n\right)}|X=x^{\left(n\right)}\right)+{\lambda ||W||}^{2}, \end{array}$$

where *n* is the number of samples, *k* is the number of landmarks, *W* represents the network parameters to minimize within regularization term to minimize and λ their weight.

In summary, we selected two architectures that alternate convolution and max-pooling layers, but whose nature is completely different. VanillaCNN presents four convolution layers and two dense layers. Dense layers are interlaid by a discrimination among the clusters previously learned from midnetwork features in a specific pose manner. RCN presents a bidirectional architecture with different branches including 3–4 convolution layers whose results are concatenated in the end of each branch to the inputs of the following one. VanillaCNN presents a descending size of filtering sizes along the network and RCN keeps it fixed.

## 5. 3D Reconstruction

**Figure 4:**
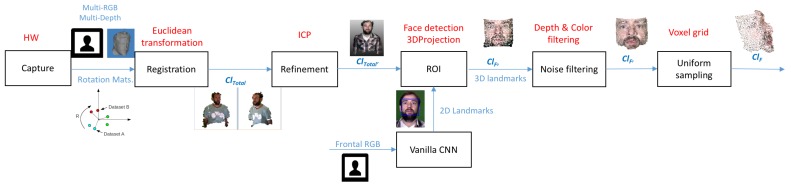
Block diagram of the different steps involved in 3D reconstruction of faces.

This section validates the algorithm developed to present one point cloud for every subject for the markers located in the scenario that are graphically shown in Figure 1 in 3DWF Dataset. The steps performed are summarized in Figure 4.

### 5.1. Registration

Clouds obtained from RGB–D devices are registered by using a rigid body transformation [38]. We use an affine transformation [39]. Ten points are selected from every cloud (two by two matching). To obtain the transformation matrix, we built an homogeneous transformation, using the frontal point cloud as reference ($Cl_{Frontal}$):

(5) $$f\left(q_{i}\right)=Rp_{i}+t$$

where *R* is the rotation matrix *R* and $t_{i}\forall i,\in \left\{1,\ldots ,3\right\}$ *t* is the translation vector. Obtaining an origin matrix with a point in every row $p_{i}=\left(x_{i},y_{i},z_{i},1\right)$ where i,∈{1,…,10} from $Cl_{Right}$ and $Cl_{Left}$ and a target matrix with a point in every row $q_{i}=\left(x_{i},y_{i},z_{i},1\right)$ where i,∈{1,…,10} from $Cl_{Frontal}$.

**Figure 5:**
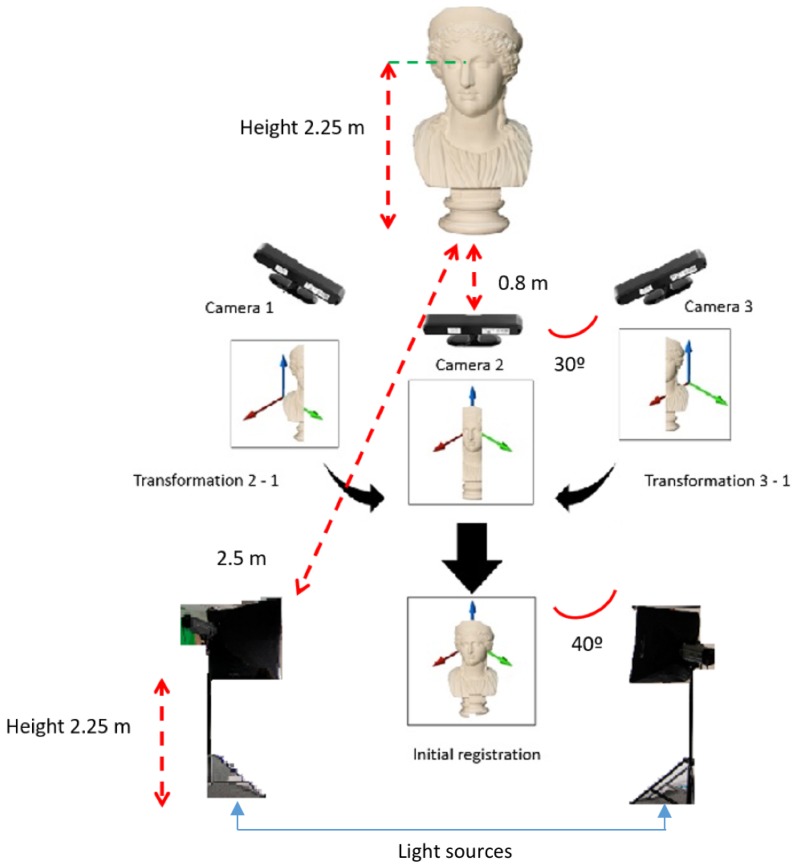
Graphical detailing of the procedure followed to reconstruct the 3D models upon the depth and RGB information captured by the three RGB–D devices.

And to overcome accuracy errors in manual annotation and obtain optimum values for $R_{left,right}$ and $T_{left,right}$ we employed Random Sample Consensus (RANSAC) [40]. Then initial clouds ($Cl_{Left}$, $Cl_{Right}$) are transformed towards the reference frontal cloud ($Cl_{Left^{\prime }}$, $Cl_{Right^{\prime }}$) and the resulting clouds are added two by two to obtain the complete cloud $Cl_{Total}$. This summation task is shown in Figure 5.

### 5.2. Refinement

The second step for reconstruction is based on Iterative Closest Point (ICP) [41] algorithm. ICP is used to minimize difference between sets of geometrical points such as segments, triangles or parametric curves. In our work, we use the point-to-point approach. Metric distance between the origin cloud ($Cl_{Left}$ and $Cl_{Right}$) and target cloud ($Cl_{Frontal}$) is minimized by the following equation:

(6) $$i=\underset{i}{arg min}∥p_{i}-q_{i}{∥}^{2},$$

where $p_{i}$ is the point belonging to the origin cloud ($Cl_{Left}$ and $Cl_{Right}$) and $q_{i}$ is a point belonging to the target cloud ($Cl_{Frontal}$). Regarding rotation and translation matrix, the algorithm iterates over the minimum square distances by:

(7) $$R,t=\sum_{i=1}^{N}\underset{R,T}{arg min}∥\left(Rp_{i}+t\right)-q_{i}{∥}^{2},$$

where *N* is the number of iterations fixed to 30 for our solution and we have also fixed the percentage of worst candidate removal to 90%. With this refinement $Cl_{Total^{\prime }}$ is obtained.

### 5.3. ROI

Once the whole cloud is built and refined, we use the Dlib face detector [42] on the RGB image from the frontal camera in order to determine the region of interest (ROI). In a similar way, we apply the face landmark detection based on VanillaCNN exposed in Section 4 obtaining the locations of facial landmarks. To project the keypoints obtained from the neural network, we use Perspective Projection Model [43]. By applying the following equations:

(8) $$X_{k}=-\frac{Z_{k}}{f}\left(x_{k}-x_{c}+\delta_{x}\right),Y_{k}=-\frac{Z_{k}}{f}\left(y_{k}-y_{c}+\delta_{y}\right),$$

where $X_{k}$, $Y_{k}$ and $Z_{k}$ are the projected coordinates in the cloud, $x_{c}$ and $y_{c}$ are the coordinates of the center of the 2D image, $x_{k}$ and $y_{k}$ are the input coordinates from the 2D image and $\delta_{x}$ and $\delta_{y}$ are the parameters to correct the distortion of the lens provided by the manufacturer. Obtaining 3D projection for the bounding box delimiters to project the cropped cloud obtained after refinement $Cl_{Total^{\prime }}$ into a cloud with mostly facial properties $Cl_{F_{0}}$.

In order to test the accuracy of $Cl_{F_{0}}$ we have considered the clouds gathered with Faro Freestyle 3D Laser Scanner ($Cl_{HD}$) as ground truth, and we have measured the average minimum distance from $\forall pt_{i}\in Cl_{F_{0}}\in Marker1$ to $\forall pt_{i}^{\prime }\in Cl_{HD}$ for every subject, obtaining as result distances in the range [16−23] mm. In addition, we should consider:

1. Since the distance from the subject to the camera is below 1 m, the error of the depth sensor should be in the range [5−15] mm according to the results exposed in [44].
2. The faces of the subjects are not rigid (although both captures have been performed on a neutral pose).

Therefore, the range measured as distance from $Cl_{F_{0}}$ to $Cl_{HD}$ proves the accuracy of the 3D reconstruction performed.

### 5.4. Noise Filtering

In this section, an algorithm to filter $Cl_{F_{0}}$ is proposed to obtain reliable face clouds. The following features are proposed:

1. **Color**. Initially we need to delimit two areas:
  (a) 2D ROI’ to extract. We have employed facial landmarks detected by VanillaCNN through data augmentation procedure presented in Section 4.2 and Section 4.1 respectively. In our setup we have detected five points: left eye (le), right eye (re), nose (*n*), left mouth (lm) and right mouth (rm). A new ROI ($ROI^{\prime }$) is defined based on a bounding box with these detections:
    $$\begin{array}{c} \left\{\left(x_{le},y_{le}\right),\left(x_{re},y_{re}\right),\left(x_{lm},y_{lm}\right),\left(x_{rm},y_{rm}\right)\right\} \end{array}$$
    $ROI_{RGB}^{\prime }$ intensities are transformed to a more uniform color space: CIELAB [45]. Components values of the two intensities samples used for thresholding are calculated in the following manner following a normal distribution:
    (9) $$\begin{array}{c} th_{i_{Lab}}=({\hat{L}}_{ROI^{\prime }}\pm w\sigma_{L_{ROI^{\prime }}},{\hat{a}}_{ROI^{\prime }}\pm w\sigma_{a_{ROI^{\prime }}},\\ {\hat{b}}_{ROI^{\prime }}\pm w\sigma_{b_{ROI^{\prime }}}) \end{array}$$
    where ${\hat{L}}_{ROI^{\prime }}$ and $\sigma_{L_{ROI^{\prime }}}$ are the mean and standard deviation of L component from CIELAB color space for the new ROI defined. Analogously for ${\hat{a}}_{ROI^{\prime }}$, $\sigma_{a_{ROI^{\prime }}}$, ${\hat{b}}_{ROI^{\prime }}$ and $\sigma_{b_{ROI^{\prime }}}$. And *w* is fixed to 0.75 in our implementation.
  (b) 3D Contour $Ct_{F_{0}}$. In this case we have defined two margins for width and height from $Cl_{F_{0}}$ to filter farther points to the cloud centroid by applying CIEDE2000 $\forall pt_{i}\in Ct_{F_{0}}$:
    (10) $$\begin{array}{c} if\Delta E_{00}^{*}\left\{\left({\hat{L}}_{ROI^{\prime }},{\hat{a}}_{ROI^{\prime }},{\hat{b}}_{ROI^{\prime }}\right),\left(L_{pt_{i}},a_{pt_{i}},b_{pt_{i}}\right)\right\}<\\ \Delta E_{00}^{*}\left(th_{1_{Lab}},th_{2_{Lab}}\right)=>pt_{i}\in Cl_{F_{FC}} \end{array}$$
    where $\Delta E_{00}^{*}$ is the metric used in CIEDE2000 and $Cl_{F_{FC}}$ is the point cloud obtained after color filtering.
2. **Depth**. Mainly focused on noise introduced by depth sensors and outliers from color filtering. For that aim we have built a confidence interval based on normal distribution of $Cl_{F_{FC}}$$\left[{\hat{Z}}_{Cl_{F_{FC}}}-w_{Z},{\hat{Z}}_{Cl_{F_{FC}}}+w_{Z}\sigma_{Z_{Cl_{F_{FC}}}}\right]$. Where $w_{Z}$ is fixed to 2.25 in our implementation.

### 5.5. Uniform Distribution

**Figure 6:**
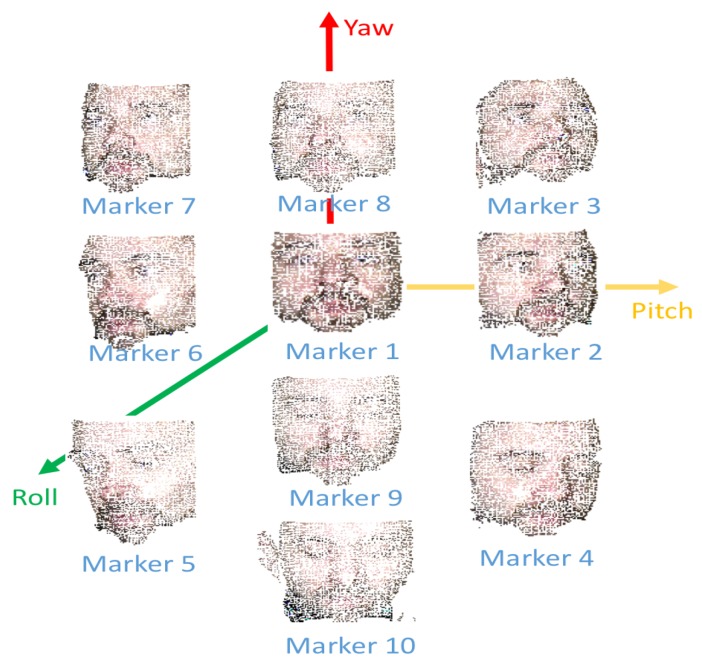
2D images extracted from the final face clouds proposed by this work.

**Figure 7:**
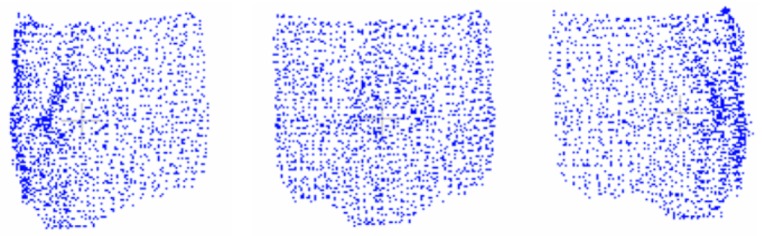
2D images extracted from the final face clouds for Marker 1 without texture mapping proposed by this work.

To provide a reliable point cloud dataset, it is important that clouds have similar resolutions and that every part of the cloud is constant regarding point-space density. For that reason, $Cl_{F_{F}}$ is divided into four parts based on its width and height. A resolution of 2K points is proposed as target resolution. Therefore, every cloud part should have 2K/4 points. An implementation of voxel grid downsampling [46] based on a dynamic radius search is used. The voxel grid filter down-samples the data by taking a spatial average of the points in the cloud through employing rectangular areas that are known as voxels. The set of points that lie within the bounds of a voxel are assigned to that voxel and will be combined into one output point. With this final step $Cl_{F}$ is composed, and sample values for one subject are displayed in Figure 6. In an analogous way one sample for Marker 1 without texture mapping is shown in Figure 7.

## 6. Head Pose Classifcation

This section describes the methods implemented to validate the alignment of head pose values of the data gathered in 3DWF dataset with markers located in the scene. We used visual information of subjects when they look at marker 1 (relaxed pose looking to the front) as reference for the other markers. Initial steps are analogous to the ones proposed in Section 5.3. In this case, we reversely used projection equations shown in (8) together with 2D Euclidean distance to gather the closest points included in $Cl_{Total^{\prime }}$ to 2D facial landmarks detected by Vanilla CNN. In this way, a new set composed by 3D facial landmarks is obtained:

$$\begin{array}{c} \{\left(X_{le},Y_{le},Z_{le}\right),\left(X_{re},Y_{re},Z_{re}\right),\left(X_{n},Y_{n},Z_{n}\right),\\ \left(X_{lm},Y_{lm},Z_{lm}\right),\left(X_{rm},Y_{rm},Z_{rm}\right)\} \end{array}$$

### Rigid Motion

Initial transformations are performed by using the Least-Squares Rigid Motion by means of SVD [47] from the set of 3D facial landmarks to obtain the corresponding rotation matrix. Let $P=p_{1},p_{2},\ldots ,p_{n}$ where $p_{i}$ are 3D coordinates of facial landmarks for marker 1 $\in R^{3}$ and $Q=q_{1},q_{2},\ldots ,q_{n}$ where $q_{i}$ are 3D coordinates of facial landmarks for markers 2–10 $\in R^{3}$ be our reference and target sets of data respectively. We are able to find a rigid transformation that optimally aligns the two sets in the least squares sense, i.e., assuming unity vector for translation matrix (subjects are static in the experiment proposed):

(11) $$\begin{array}{c} R=\underset{R\in SO(3)}{\mathrm{argmin}}\sum_{i=1}^{n}w_{i}||Rp_{i}-q_{i}{\left|\right|}^{2} \end{array}$$

By Restating the problem so that the translation would be zero, and simplifying the expression we cand reformulate the problem:

(12) $$\begin{array}{c} ||Rp_{i}-q_{i}{\left|\right|}^{2}==tr\left(WY^{T}RX\right) \end{array}$$

where $W=diag(w_{1},\ldots ,w_{n})$ is an nxn diagonal matrix with the weight $w_{i}$ on diagonal entry *i*, *Y* is the dxn matrix with $y_{i}$ as its columns and *X* is the dxn matrix with $x_{i}$ as its columns. tr is the trace of a square matrix (sum of the elements on the diagonal) and owes commutative property with respect to product. Therefore we are looking for a rotation *R* that maximizes $tr(RXWY^{T})$. Now we have denoted dxd covariance matrix $S=XWY^{T}$. If we take Single Value Decomposition (SVD) of *S* such that $S=U\sum V^{T}$ relying on the fact that that *V*, *R* and *U* are all orthogonal matrices, so $V^{T}RU$ is also an orthogonal matrix and we can assume identity. Therefore we can calculate corresponding rotation matrix in the following way:

(13) $$\begin{array}{c} R=VU^{T} \end{array}$$

Given a rotation matrix *R*, we can compute the Euler angles, ϕ, θ, ψ by equating each element in R with the corresponding element in the matrix product $R_{Z}\left(\phi \right)R_{Y}\left(\theta \right)R_{X}\left(\psi \right)$. This results in nine equations that have been used to find the Euler angles.

## 7. Results

In this subsection, metrics used for evaluation and results obtained by them for the proposed architectures and datasets are presented.

### 7.1. Facial Landmark Detection

#### 7.1.1. Training Details

Initially, we have defined training details for every dataset:

1. **3DU Dataset**. Number of viewpoints augmented to split dataset, number of subjects included per view and learning rate base for 3DU Dataset are expressed in Table 1.
  Subjects are randomized for every viewpoint based on splits presented in Section 4.1.
2. **AFLW**. Number of images to split dataset and learning rate base for AFLW Dataset are shown in Table 2.

#### 7.1.2. Error Metric

The euclidean distance between the true and estimated landmark positions normalized by the distance between the eyes (interocular distance) is used:

(14) $$\begin{array}{c} \epsilon =\frac{1}{KN}\sum_{n=1}^{N}\sum_{k=1}^{K}\sqrt{\frac{{\left(w_{k}^{n}-{\hat{w}}^{\left(n\right)}\right)}^{2}+\left(h_{k}^{\left(n\right)}\right)-\left({\hat{h}}_{k}^{\left(n\right)}\right)}{D\left(n\right)}} \end{array}$$

where *K* is the number of landmarks (5 in our work), *N* is the total number of images, D(n) is the interocular distance in image (n). $\left(w_{k}^{n},h_{k}^{\left(n\right)}\right)$ represent the true and $\left({\hat{w}}_{k}^{n},{\hat{h}}_{k}^{\left(n\right)}\right)$ estimated coordinates for landmark k in image n, respectively. Localization error is measured as a fraction of the inter-ocular distance, a measure invariant to the actual size of the images. We declare a point correctly detected if the pixel error is below 0.1 interocular distance.

#### 7.1.3. Accuracy

**Figure 8:**
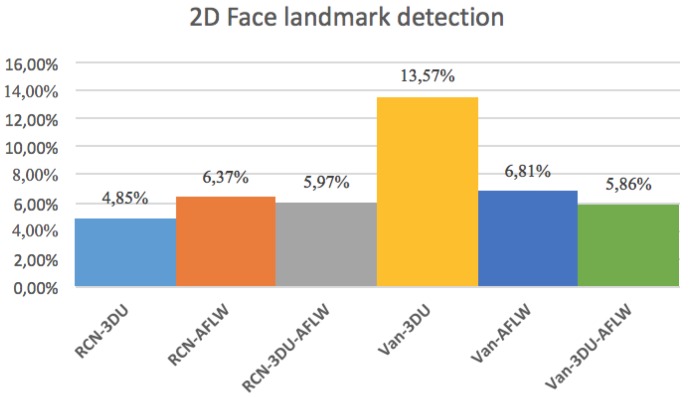
Results obtained for both pipelines of face landmark detection with different combinations of datasets.

The histogram in Figure 8 show that the error for the different combinations of networks, explained in Section 4.2 and datasets. It can be derived that the lowest error rate is by training RCN with the 3DU dataset.

Massive normalized 2D projections of this dataset learned in a bidirectional approach reduces the error. In addition, we mention that the network layers based on midnetwork features proposed by VanillaCNN achieve the worst results with the same training and testing data since this is an specific solution for another nature of data, but weights and bias pre-learned from 3DU dataset increases the performance of the algorithm, and helps to achieve the best results for AFLW after finetuning. In this case, massive data initialize properly midnetwork features so that the network can go beyond in global minimum target for common datasets of landmark detection such as AFLW. This procedure is shown, using the visualization of filters, in Table 3 and Table 4 for last convolution and max-pooling layers, where brighter color intensities represent stronger activations. It can be derived that the initial training of 3DU provides blunter features at this stage of the learning due to triangulation procedure to gather mesh input data. It can be inferred as well that the fine-tuning process provides sharper features that increase the performance of the network.

### 7.2. Head Pose Classification

**Figure 9:**
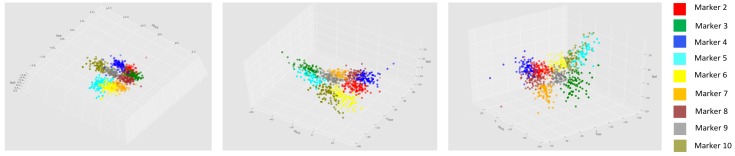
Graphical plots of Euler Angles obtained for the different markers of 3DWF.

To validate the proposed dataset, we assumed Euler angles calculated previously as head pose angles (pitch (ϕ), yaw (ψ) and roll (θ)). Three 2D projections of this data can we noted on Figure 9. It can be inferred that the distribution of the data is quite uniform.

#### Accuracy

Two validation methods have been tested in order to classify the Euler angles calculated for the projection of the facial andmarks obtained by fine tuning the initial training of VanillaCNN with the data augmentation procedure exposed in Section 4.1 with the training of AFLW. The methods selected are Linear Discriminant Analysis (LDA [48]) and Gaussian Naive Bayes (GNB [49]). The aim of those classification methods is to validate the data capture, and the projection of the facial landmarks estimated to the point clouds gathered. The results and the main features of the proposed methods can be noticed on Table 5.

**Figure 10:**
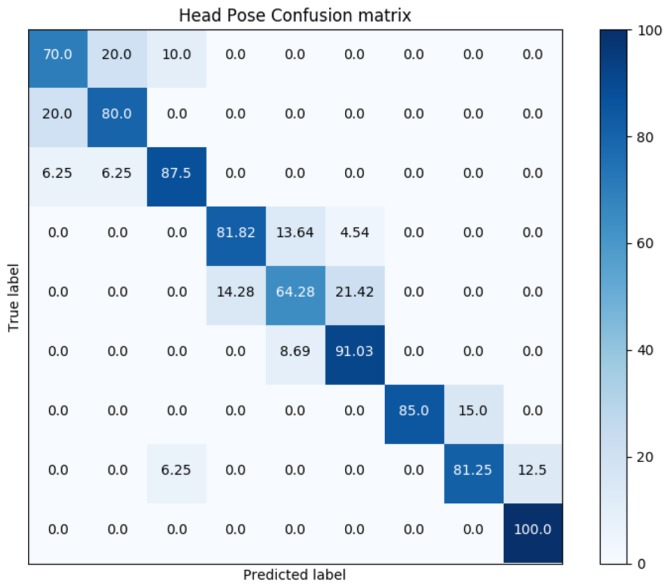
Confusion Matrix calculated for Head Pose validation method.

Confusion matrix for GNB classification, where rows correspond to ground truth markers (2–10) and columns to predicted markers in the same range, is shown in Figure 10. Results show promising values for a simple classification technique such as GNB. It can be noticed that those markers where the subjects are looking to one side of the scene (such as 2 or 5) are the most complex to predict, and those markers where the subjects are looking straight and modifying their pitch (such as 10) the simplest.

## 8. Conclusions

In this paper, we have presented an optimized multi-camera RGB–D system for facial properties to capture accurate and reliable data. In this scope we have performed a data collection including 92 people fulfilling the need of a 3D facial dataset able to exploit capabilities of deep learning paradigm in 3D scope. In addition, we provide a complete pipeline to process data collected and pose a challenge for Computer Vision and Machine Learning research community by annotating human characteristics such as age or gender. The collected RGB–D streams allow other related tasks such as face tracking or 3D reconstruction with a wide source of visual information that increase the performance of common acquisition systems for extreme head poses.

In this scope, we found facial landmark detection one of the main tasks where our work should contribute to research lines that project 3D information into a more feasible and less costly domain such as 2D. For that reason, we have proposed an innovative data augmentation method, tested and discussed its accuracy on two state-of-the-art deep learning solutions. We have trained and evaluated synthetic and visual imaging data on two complementary architectures, finding a combined solution that enhances results for a very common deep net architecture like Vanilla CNN.

Finally, the alignment of the path proposed to the subjects by ten markers is validated by implementing a geometric approach for head-pose through previously estimated features. The refinement of the learning techniques implemented for this task is one of the lines of research proposed for future work.

## Figures and Tables

**Table 1:** Parameters for training 3DU dataset.

Dataset	Subjects		Learning Rate	
	Train.	Val.	RCN	Vanilla
3DU	35	12	$10^{-4}$	$10^{-4}$

**Table 2:** Parameters for training AFLW dataset.

Dataset	Images			Learning Rate	
	Train	Val.	Test	RCN	Vanilla
AFLW	9000	3000	1000	$10^{-4}$	$10^{-4}$

**Table 3:** Representation of the last layer of convolution from VainillaCNN.

Conv 3DU	Conv 3DU + AFLW
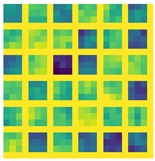	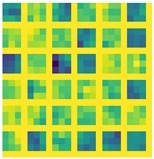

**Table 4:** Representation of the last layer of max-pooling from VainillaCNN.

Pool 3DU	Pool 3DU + AFLW
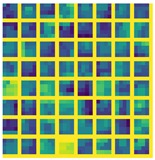	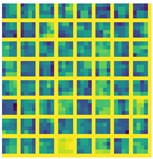

**Table 5:** Results for head pose classification.

Method	Training Samples	Testing Samples	Training Accuracy	Testing Accuracy
LDA	80%	20%	82%	83%
GNB	80%	20%	84%	84%

## Abbreviations

The following abbreviations are used in this manuscript:

RGB-D	Red Green Blue-Depth
3DWF	3D Wide Faces
Multi-PIE	Multi Pose Illumination Expression
3DU	3DUniversum
3DMM	3D Morphable Model
MRF	Markov Random Field
CNN	Convolutional Neural Network
USB	Universal Serial Bus
LED	Light-Emitting Diode
STD	Standard Deviation
MAD	Median Absolute Deviation
AFLW	Annotated Facial Landmarks in theWild Dataset
RCN	Recombinator Networks
ReLU	Rectifier Linear Unit
RANSAC	Random Sample Consensus
ICP	Iterative Closest Point
ROI	Region Of Interest
CIELAB	color space defined by the International Commission on Illumination (CIE)
CIEDE2000	color difference defined by the International Commission on Illumination (CIE)
SVD	Single Value Decomposition
LDA	Linear Discriminant Analysis
GNB	Gaussian Naive Bayes

## Appendix A. Light Source

**Table A1:** Main features of the light source employed in 3DWF setup.

Feature	Value
Power	36 W
Color temperature	5800 K ± 300 K
Luminous flux	4200 lm

## Appendix B. Quantitative Evaluation of 3DWF Set-Up

### Appendix B.1. 3DWF Distance from Model to the Frontal Camera

**Table A2:** Evaluation of the distance from the model to the frontal camera.

Distance (cm)	Cloud Points
80	7754
100	5871
120	4342

### Appendix B.2. 3DWF Light Source Luminous Flux

**Table A3:** Evaluation of the influence of the light source luminous flux.

Lm	Points of the Cloud		
	Cloud Cam. 1	Cloud Cam. 2	Cloud Cam. 3
5000	5859	7693	7460
2625	6029	7896	7986
1435	6084	8221	7562
250	6621	8841	8935

### Appendix B.3. 3DWF Light Source Orientation

**Table A4:** Evaluation of the orientation of the light sources.

Focus	Angles		Cam 1		Cam 2		Cam 3	
	Light	Cams	$\tilde{I}$	MAD	$\tilde{I}$	MAD	$\tilde{I}$	MAD
Front	30${}^{\circ }$	40${}^{\circ }$	189	56	87	58	124	73
Side	40${}^{\circ }$	30${}^{\circ }$	138	51	89	51	128	62
Front	30${}^{\circ }$	60${}^{\circ }$	164	50	100	68	122	81

### Appendix B.4. 3DWF Cameras Orientation

**Table A5:** Evaluation of the orientation of the cameras.

Angle	Cam 1		Cam 2		Cam 3	
	$\bar{I}$	$\sigma_{I}$	$\bar{I}$	$\sigma_{I}$	$\bar{I}$	$\sigma_{I}$
30${}^{\circ }$	129.35	62.69	94.22	66.38	118.35	72.43
40${}^{\circ }$	172.00	71.09	98.29	71.37	122.51	75.80
60${}^{\circ }$	171.50	71.90	108.674	79.94	124.57	85.75
